# Investigation of the relationship between pulmonary lesions based on lung ultrasound and respiratory clinical signs in foals with suspected pulmonary rhodococcosis

**DOI:** 10.1038/s41598-023-46833-2

**Published:** 2023-11-08

**Authors:** Alicja Rakowska, Michał Czopowicz, Andrzej Bereznowski, Lucjan Witkowski

**Affiliations:** Division of Veterinary Epidemiology and Economics, Institute of Veterinary Medicine, Warsaw University of Life Sciences–SGGW, Nowoursynowska 159C, 02-776 Warsaw, Poland

**Keywords:** Zoology, Diseases, Medical research, Risk factors, Signs and symptoms

## Abstract

*Rhodococcus equi* is a widely recognized bacterium responsible for pneumonia in preweaned foals. On endemic farms, foals with a subclinical course of the disease usually outnumber those presenting clinical signs. The disease is typically chronic and mainly manifests as fever and dyspnoea. Currently, field diagnosis is often based on lung ultrasound (LUS); however, both diagnostic and therapeutic approaches vary among practitioners and considerably change over time. This longitudinal, prospective study was designed to describe the appearance and progression of rhodococcal pulmonary lesions during the first months of life based on LUS and to evaluate the relationship between the presence and severity of rhodococcal pulmonary lesions and the occurrence of respiratory clinical signs in foals from farms with endemic *R. equi* infections. Nearly 26% of foals demonstrated respiratory signs highly suggestive of pulmonary rhodococcosis, and approximately 70% of the foals had abnormalities detected on LUS without concurrent clinical signs. The appearance and development of LUS abnormalities were age-related. An abscess diameter exceeding 15 mm in LUS and other pleural lesions were significantly linked with the occurrence of clinical signs suggestive of pulmonary rhodococcosis (P < 0.001) and may be considered predictive factors of rhodococcal pneumonia in foals.

## Introduction

*Rhodococcus equi* is an opportunistic, ubiquitous, intracellular, gram-positive pathogen responsible for pyogranulomatous pneumonia in preweaned foals^1,2^. The disease mostly occurs sporadically; however, some breeding farms suffer from endemic rhodococcosis, possibly due to favourable environmental and management conditions^1,2^. The main clinical signs are fever, lethargy and decreased appetite, ill-thrift, intermittent cough, and respiratory distress. Rhodococcal pneumonia usually takes a chronic, rarely subacute or acute course, with a large proportion of subclinical cases^3–5^.

The increasing popularity of lung ultrasound (LUS) screening of foals before weaning has improved the ability to identify foals with subclinical pulmonary rhodococcosis in equine practice. The highest prevalence of LUS abnormalities was estimated at 92% and described in 2010 in one of the German breeding farms^3^ and 95% in southern Brazil^4^; however, the relationship between LUS abnormalities and the risk of developing clinical pneumonia has not been established. To date, most of the clinical studies on *R. equi* infection in foals are focused on establishing diagnostic criteria for treatment implementation^6^. Two scores were proposed to evaluate the severity of LUS abnormalities and recommend therapy: total maximal diameter (TMD) based on the total diameter of all detected lesions^6,7^ and the Slovis score based on the maximal diameter of the largest detected lesion^5^. Recommendations for therapy considering both scores have changed significantly over the past decade. Nevertheless, in many countries, the most common practice in field settings remains to start treatment at the onset of clinical signs^8–10^.

Epidemiological data regarding rhodococcosis are still limited and are mainly restricted to a few studies from large equine breeding farms. In studies from Texas, 17% of foals were diagnosed as clinically affected, 63% as subclinical cases, and 20% as unaffected^5^. In a very recent paper including 5 farms over 3 breeding seasons in North America, the cumulative incidence of clinical rhodococcosis was estimated at 5%^11^. In another study, 27% of foals on a large American Quarter Horse farm in Texas were diagnosed with rhodococcal pneumonia, with a mortality rate of 4%^8^. The previous epidemiological data from 12 stud farms in central and eastern Poland estimated the clinical prevalence at 5–10% and the mortality rate at 3–7%^12^. Considerable seasonal variations and very divergent morbidity and mortality in endemic farms are observed.

Our study was designed to (i) investigate the progression of rhodococcal pulmonary lesions in foals during the first 4 months of life, (ii) evaluate the relationship between the appearance and presence of rhodococcal pulmonary lesions in LUS examination and the occurrence of respiratory clinical signs, and (iii) evaluate the diagnostic potential of clinical signs in pulmonary rhodococcosis.

## Materials and methods

### Study population

The study was carried out during 3 breeding seasons from 2019 to 2021, and foals born on 5 Polish farms (A to E, 4 national studs and one private horse farm) were enrolled. Two farms (A and B) participated in the study for the entire period: foals from one were enrolled during two breeding seasons (C in 2019 and 2020), and foals from two were enrolled during one breeding season (D in 2020 and E in 2021). Four farms (A–D) were considered endemic based on the history of multiple *R. equi* infections confirmed either by postmortem sampling (mostly) or bronchoalveolar lavage sampling and positive microbiological cultures during over a decade of cooperation with the university *(since 2002). The endemic status of the fifth farm has not yet been confirmed.

The study population included 185 foals, 87 colts (47.0%) and 98 fillies (53.0%), of 3 breeds—85 Pure Arabians (45.9%), 63 Polish Anglo-Arabians (34.1%), and 37 Thoroughbreds (20.0%). The sex distribution did not differ between farms (P = 0.245). Four farms included only one breed of horses, and one farm consisted of 2 breeds: Pure Arabians and Polish Anglo-Arabians (Table 1). The study population was considered representative of the Polish population of foals aged up to 4 months kept in studs affected by pulmonary rhodococcosis.

**Table 1:** Characteristics of foals in the study population.

Stud	Number of foals (% from the study population)	Number (%) of colts	Number (%) of foals of a certain breed			Epidemiological status of the stud
			Pure Arabians (oo)	Thoroughbreds (xx)	Polish Anglo-Arabians	
A	67 (36.2)	36 (54)	61 (91)	0	6 (9)	Endemic
B	57 (30.8)	25 (44)	0	0	57 (100)	Endemic
C	25 (13.5)	9 (36)	0	25 (100)	0	Endemic
D	24 (13.0)	9 (38)	24 (100)	0	0	Endemic
E	12 (6.5)	8 (67)	0	12 (100)	0	Unknown
Total	185	87 (47.0)	85 (46.0)	37 (20.0)	63 (34.0)	

Three farms (A–C) participated in a voluntary screening program established by the Department of Veterinary Epidemiology and Economics (Institute of Veterinary Medicine, SGGW, Poland) with the objective of improving and promoting noninvasive and widely available field identification of foals affected by clinical or subclinical pulmonary rhodococcosis based on clinical and LUS examination. All the participating farms practised internal parasite control and followed vaccination protocols against infections with equine influenza virus and equine herpesvirus type 1 and 4 in pregnant mares.

### Screening schedule and procedures

The foals were examined approximately every two weeks between the 2nd week and 4th month of life. In total, 797 examinations were performed; however, the number of examinations each foal underwent varied considerably, with 107 foals (57.9%) examined 5 to 7 times, 35 foals (18.9%) examined 3 or 4 times, and 43 foals (23.2%) examined only once or twice (Table S1). The number of examinations of individual foals varied due to practical problems with cooperation between the stable personnel, stable attending veterinarian, stable manager, and veterinarian skilled in LUS. The median (interquartile range [IQR]) age at the first examination was 29 (23 to 47) days, with a range from 12 to 111 days. The detailed number of foals examined at each age is presented in Table S2.

Each screening procedure was performed by the same veterinarian and comprised three elements: (i) clinical history including expected foaling date, duration of gestation, observed or assisted parturition, and foal illness during the first week of life; (ii) physical examination including rectal temperature, the appearance of the mucous membranes and capillary refill time, palpation of the submandibular lymph nodes, heart rate, respiratory rate, general respiratory pattern, and thoracic auscultation; and (iii) LUS examination.

Based on our clinical experience, foals were suspected of *R. equi* pneumonia if they demonstrated at least one of the following clinical signs (henceforth referred to as respiratory clinical signs): (i) fever > 39.5 °C provided that other apparent potential causes of fever were ruled out; (ii) dyspnoea defined as a respiratory rate > 30 breaths per minute accompanied by a noticeable increase in the respiratory effort; and (iii) marked murmurs or crackles on thoracic auscultation^13^. The decision to include fever as a diagnostic criterion for *R. equi* pneumonia was based on the endemic status of the farms and previously documented association of pyrexia with *R. equi* pneumonia in foals at a certain age^7,11,14^.

LUS examination was performed using a portable ultrasound scanner (4Vet Slim, Dramiński, Gietrzwałd, Poland) with a linear 8–10 MHz transducer (Dramiński, Gietrzwałd, Poland), the type of transducer most suited to the evaluation of pleura and superficial pulmonary lesions^14^.

During the examination, foals were restrained by stable personnel. No sedation was used; however, examination of some foals needed using a twitch. Hair was not clipped, and the chest was drenched with alcohol. Each intercostal space was examined dorsoventrally, and the transducer was moved forward from the 17th intercostal space to the area of the musculus triceps brachii (approximately the 5–6th intercostal space). Three types of LUS abnormalities were defined, observed and analysed as follows: first, multiple B-lines, defined as hyperechoic, vertical artefacts originating from horizontal pleural line, visible simultaneously on the screen and are hard to count due to their considerable number and the dynamic type of ultrasonographic visualization; second, the presence of irregular or thickened lining of the pleural surface and/or free pleural fluid (referred to as other pleural lesions); third, the presence, number, and diameter of suspected lung abscesses were defined as focal hypoechoic areas of consolidation within the lung. If multiple abscesses were present, the maximum diameter of the largest abscess was used in further analyses.

### Treatment of foals suspected of clinical *R. equi* pneumonia

During our study, antimicrobial therapy was implemented based on the individual veterinarian’s decision (ours or the attending veterinarian of the stud) given the presence of respiratory clinical signs at the moment of examination (or in a few preceding days as reported by foals’ caretakers), the presence of LUS abnormalities and endemic status of the stud. In total, 53/185 foals (28.7%) were treated based on the aforementioned criteria: 15/67 (22.4%) in Stable A, 28/57 (49.1%) in stable B, 2/25 (8.0%) in stable C, 6/24 (25.0%) in stable D, and 2/12 (16.7%) in stable E. Recommended antimicrobial treatment during the study included the combination of rifampicin (5 mg/kg p.o. BID) and clarithromycin (7.5 mg/kg p.o. BID) or tulathromycin (2.5 mg/kg i.m. weekly)^13,15,16^. The duration of a particular treatment depended on the clinical presentation and presence of LUS findings; however, the duration was never shorter than 5 weeks.

### *R. equi* isolation

Clinical specimens taken from diseased or dead foals were submitted to bacteriological examination in the Microbiological Diagnostic Laboratory, Institute of Veterinary Medicine, Warsaw University of Life Sciences-SGGW. Samples were cultured on Columbia Agar supplemented with 5% sheep blood (Graso, Poland) and selectively modified CAZ-NB medium incubated for 48 h at 37 °C in aerobic conditions^17^. *R. equi* isolates were recognized based on their growth and cell morphology as well as biochemical characteristics using the API Coryne test (bioMérieux, France). To confirm the isolate identification, the CAMP test with *Staphylococcus aureus* ATCC 25923 was performed. Additionally, the presence of two *R. equi* genes, *choE* and *vapA*, was determined by PCR as previously described^17^. Antimicrobial susceptibility testing for *R. equi* isolates was performed according to the guidelines of the Clinical and Laboratory Standards Institute^18^. None of the *R. equi* strains isolated from the foals that died during our study were found to be resistant to the recommended drugs.

### Statistical methods

Categorical variables are presented as counts and percentages and were compared between groups using the maximum likelihood G-test. The age-related prevalence was calculated as a ratio between the number of foals affected by a given condition at a particular age (defined as 2-week age classes: 1–2 weeks, 3–4 weeks, etc.) and the total number of foals examined at this age. The 95% confidence intervals (CI 95%) for percentages were calculated using Wilson’s score method^19^. Numerical variables were assessed with respect to the normality of distribution using histograms and the Shapiro‒Wilk W test. As normality of distribution was violated in most cases, numerical data were summarized using the median, interquartile range (IQR), and range and compared between groups using the Mann‒Whitney *U* test.

The relationship between the presence of various LUS abnormalities (the explanatory variables) and the occurrence of clinical signs suggestive of rhodococcal pneumonia (the outcome variable) was evaluated using the mixed-effect binary logistic regression model according to the following formula:

$$\mathrm{P}\left(\mathrm{Y}=1\right)=\frac{1}{1+{\mathrm{e}}^{-({\mathrm{B}}_{0}+{\mathrm{B}}_{\mathrm{age}}\times {\mathrm{X}}_{\mathrm{age}}+{\mathrm{B}}_{\mathrm{b}}{\times \mathrm{X}}_{\mathrm{b}}+{{\mathrm{B}}_{\mathrm{PL}}\times {\mathrm{X}}_{\mathrm{PL}}+\mathrm{B}}_{\mathrm{LA}}\times {\mathrm{X}}_{\mathrm{LA}}+{\mathrm{B}}_{\mathrm{S}}{\times \mathrm{X}}_{\mathrm{S}}+{\mathrm{B}}_{\mathrm{BL}}{\times \mathrm{X}}_{\mathrm{BL}}+F+S+D)}}$$

where P(Y = 1) is the probability of the occurrence of respiratory clinical signs and B_0_ is an intercept. Three variables were fitted as random effects and forced into the model: the foal (*F*) to control for the various number of times the same foal was examined (paired data), the stud (*S*) to control for the environmental conditions in the stud from which a foal came, and the year (*D*) to control for different microclimatic conditions in subsequent years^20^. Six variables were fitted as fixed effects: the age at which a foal was examined (X_age_), which was expressed as 2-week intervals and forced into the model to control for the dependent nature of data associated with the longitudinal design of this study; the breed (X_b_; binary variable: Pure Arabians or Polish Anglo-Arabians vs. Thoroughbreds^21^) to control the potential breed-related susceptibility to rhodococcosis; the presence of other pleural lesions (X_PL_, binary variable: absent vs. present), the number of lung abscesses (X_LA_, binary variable: none vs. ≥ 1), the maximum diameter of the largest lung abscess (X_S_, binary variable: < 15 mm vs. ≥ 15 mm), and the occurrence of multiple B-lines (X_BL_, binary variable: absent vs. present). The variables corresponding to the LUS abnormalities were included first individually (univariable analysis) and then *en bloc* and eliminated from the model according to the backwards stepwise procedure (multivariable analysis).

The relationship between the presence of various clinical signs (the explanatory variables) and the presence of lung abscesses in LUS, considered as a basis for the presumptive diagnosis of rhodococcal pneumonia (the outcome variable), was evaluated using the mixed-effect binary logistic regression model according to the following formula:

$$\mathrm{P}\left(\mathrm{Y}=1\right)=\frac{1}{1+{\mathrm{e}}^{-({\mathrm{B}}_{0}+{\mathrm{B}}_{\mathrm{age}}\times {\mathrm{X}}_{\mathrm{age}}+{\mathrm{B}}_{\mathrm{b}}{\times \mathrm{X}}_{\mathrm{b}}+{{\mathrm{B}}_{\mathrm{BT}}\times {\mathrm{X}}_{\mathrm{BT}}+\mathrm{B}}_{\mathrm{HR}}\times {\mathrm{X}}_{\mathrm{HR}}+{\mathrm{B}}_{\mathrm{MM}}{\times \mathrm{X}}_{\mathrm{MM}}+{\mathrm{B}}_{\mathrm{LN}}{\times \mathrm{X}}_{\mathrm{LN}}+{\mathrm{B}}_{\mathrm{D}}{\times \mathrm{X}}_{\mathrm{D}}+{\mathrm{B}}_{\mathrm{A}}{\times \mathrm{X}}_{\mathrm{A}}+F+S+D)}}$$

where P(Y = 1) was the probability of the occurrence of lung abscess in LUS and B_0_ was an intercept. The above three variables (foal (*F*), stud (*S*), and year (*D*)) were fitted as random effects, while the age at which a foal was examined (X_age_) and breed (X_b_) were fitted as fixed effects. These 5 variables were forced into the model. The following six variables corresponding to clinical signs were fitted as fixed effects, entered into the model, and eliminated according to the backwards stepwise procedure (multivariable analysis): body temperature (X_BT_, numerical variable), heart rate per minute (X_HR_, numerical variable), mucous membranes (X_MM_: 3-class nominal variable: pink and moist vs. pale vs. congested), lymph nodes (X_LN_; 3-class nominal variable: normal vs. unilaterally enlarged vs. bilaterally enlarged), dyspnoea (X_D_; binary variable: absent vs. present), and thoracic auscultation (X_A_; 3-class ordinal variable: normal vs. mild murmurs and crackles vs. marked murmurs and crackles).

The relationships between variables were described using crude odds ratios (ORs) in the univariable analysis and adjusted ORs (OR_adj_) in the multivariable analysis. The cutoff values for numerical variables (the number of abscesses and the size of the largest abscess) were determined using area under the receiver operating characteristic curve (AUROC) analysis based on maximizing Youden’s index (J). For the optimal cutoff value, diagnostic sensitivity (Se) and specificity (Sp) as well as positive (LR+) and negative (LR−) likelihood ratios were calculated. The significance level (α) was set at 0.05, and all statistical tests were two-tailed. Data were analysed in TIBCO Statistica 13.3 (TIBCO Software Inc., Palo Alto, CA) and IBM SPSS Statistics 28.0 (IBM Corporation, Armonk, NY).

### Ethics

The study was carried out following the standards recommended by The Act of the Polish Parliament of 15 January 2015 on the Protection of Animals Used for Scientific or Educational Purposes (Journal of Laws 2015, item 266). According to Polish legal regulations (The Act of the Polish Parliament of 15 January 2015 on the Protection of Animals Used for Scientific or Educational Purposes, Journal of Laws 2015, item 266), no formal ethics consent was required for this study except for the informed consent for participation in the study, which was obtained from the person responsible for each of the studs.

### Informed consent

All of the representatives responsible for animals involved in the study were informed in advance about the intended publication of the collected results.

## Results

### Occurrence of respiratory clinical signs and LUS abnormalities in foals

**Figure 1:**
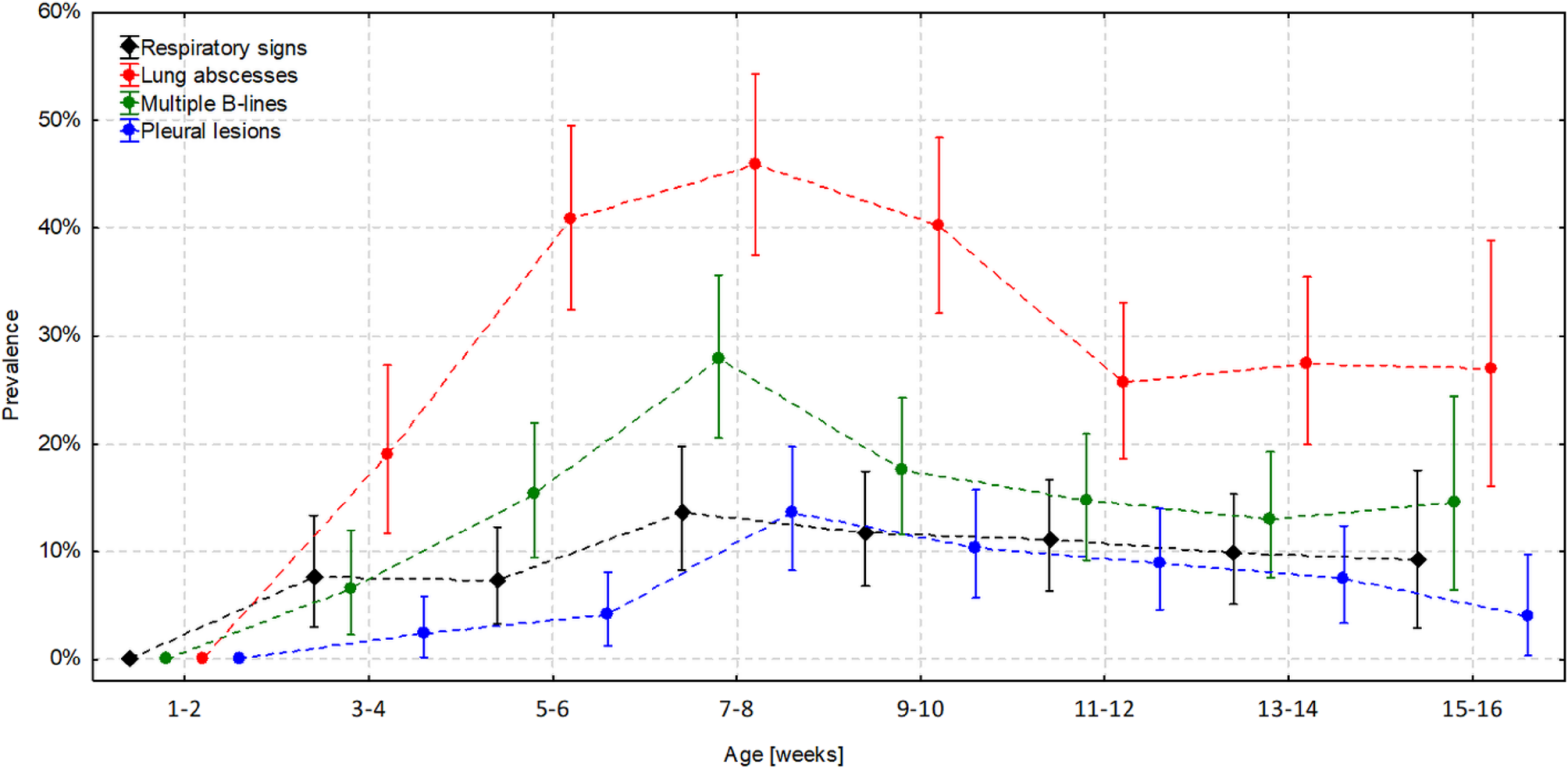
Age-related occurrence (with 95% confidence intervals) of respiratory clinical signs and lung ultrasound (LUS) abnormalities in foals.

Respiratory clinical signs were observed in 48/185 foals—25.9% (CI 95% 20.2–32.7). Marked murmurs or crackles on auscultation were the most frequent findings (20.0%, CI 95% 14.9–26.3%; 37/185 foals), followed by fever (14.6%, CI 95% 10.2–20.4%; 27/185 foals) and dyspnoea (3.8%, CI 95% 1.8–7.6%; 7/185 foals). Respiratory clinical signs were observed in a significantly higher percentage of foals in stud B (51%) than in the remaining 4 studs, in which 12% to 20% showed respiratory signs (*P* < 0.001) (Fig. 1). Body temperature ranged from 36.9 to 41.5 °C, with a median (IQR) of 38.3 °C (38.1–38.6 °C). The heart rate ranged from 48 to 168 bpm, with a median (IQR) of 80 (72–92) bpm. Enlargement of lymph nodes was observed in 84/185 foals (45.4%; CI 95% 38.4–52.6%), of which 50 foals had bilateral lymph node enlargement.

LUS abnormalities were observed in 130/185 foals (70.3%; CI 95% 63.3–76.4%). Lung abscesses were found significantly more often (61.6%, CI 95% 54.4–68.3%; 114/185 foals) than multiple B-lines (40.5%, CI 95% 33.7–47.7%; 75/185 foals) and other pleural lesions (20.5%, CI 95% 15.3–26.9%; 38/185 foals). In total, B-lines were observed in 174/185 foals (94.1%, CI 95% 89.7–96.6%). An increased amount of pleural fluid was detected only in 7 foals (3.8%, CI 95% 1.8–7.6%). The percentage of foals with LUS abnormalities did not differ significantly between studs (*P* = 0.248), and in all studs, LUS abnormalities were observed significantly more often than respiratory signs (Fig. S1).

The number of lung abscesses detected in a foal during a single LUS examination ranged from 1 to 14, with a median (IQR) of 2 (1–3). The maximum diameter of the largest lung abscess found in a foal ranged from 2 to > 50 mm, with a median (IQR) of 20 (10–34) mm.

### Development of rhodococcal pulmonary lesions in foals during the first 4 months of life

The age-related prevalence of respiratory signs ranged from 6 to 13% and did not change significantly in the study period (*P* = 0.659). In contrast, the prevalence of lung abscesses (*P* < 0.001), multiple B-lines (*P* = 0.004), and other pleural lesions (*P* = 0.048) was significantly associated with age (Fig. 1). The number of these abnormalities increased significantly in the first weeks of life and peaked at the age of 7–8 weeks. At this age, the occurrence of lung abscesses was 45.8% (CI 95% 37.5–54.3%), which was significantly higher than that of multiple B-lines (27.5%, CI 95% 20.6–35.7%) and other pleural lesions (13.0%, CI 95% 8.3–19.8%). After this timepoint, the prevalence of LUS abnormalities decreased gradually and stabilized in the 4^th^ month of life at 25%, 12–14%, and 7–8%, respectively. The percentage of foals with lung abscesses was 2.4-fold (11–12 weeks old) to 6.3-fold (5–6 weeks old) higher than that of foals demonstrating respiratory signs. Detailed data on age-related prevalence are presented in Table S3.

### Relationship between the appearance and progression of LUS abnormalities and the occurrence of respiratory clinical signs

Controlled for the random effect of the foal, the stud, and the year in which the foal was born and the fixed effect of the breed and age at the time of examination, the occurrence of respiratory signs was significantly associated with the presence of lung abscesses (OR 6.10, CI 95% 3.43–10.9; *P* < 0.001), other pleural lesions (OR 8.14, CI 95% 3.89–17.1; *P* < 0.001), and multiple B-lines (OR 3.03, CI 95% 1.68–5.46; *P* < 0.001) in the univariable analysis.

**Figure 2:**
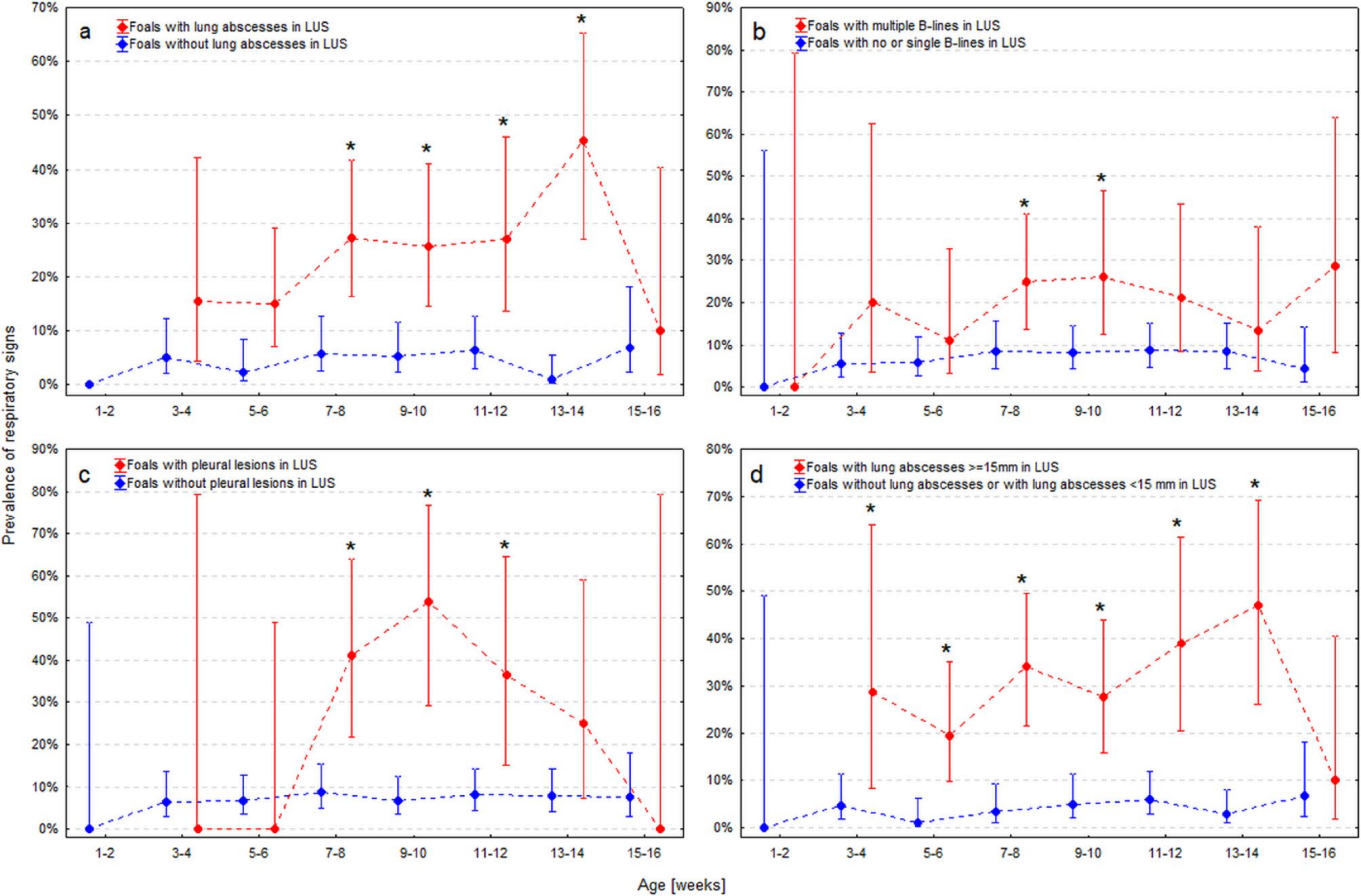
(**a**–**d**) Age-related prevalence of respiratory clinical signs (with 95% confidence intervals) in foals with and without: lung abscesses (**a**), multiple B-lines (**b**), other pleural lesions (**c**), and lung abscesses ≥ 15 mm in the lung ultrasound (LUS) examination. Asterisks (*) indicate difference in the prevalence of respiratory clinical signs between foals with and without abnormalities detected on LUS at the same age (P < 0.05).

Compared to foals without lung abscesses, the prevalence of respiratory signs was significantly higher in 7- to 8-week-old foals with lung abscesses and remained significantly elevated until 15–16 weeks of age (Fig. 2a). Two numerical characteristics of lung abscesses were significantly positively linked with the occurrence of respiratory signs: the number of abscesses (OR 1.52, CI 95% 1.33–1.74; *P* < 0.001) and the maximum diameter of the largest lung abscess (OR 1.07, CI 95% 1.06–1.09; *P* < 0.001). Based on the AUROC analysis, the optimal cutoff value for the discrimination between foals with and without respiratory signs was ≥ 1 lung abscess (AUROC 75.2%, CI 95% 68.8–81.5%) and maximum diameter of the largest lung abscess of ≥ 15 mm (AUROC 78.3%, CI 95% 71.7–84.8%).

Compared to foals without B-lines or with only a few B-lines (which we usually call 3–5 B-lines visible on the screen) during the LUS, the prevalence of respiratory signs was significantly higher in foals with multiple B-lines at the age of 7–8 weeks (*P* = 0.012) and 9–10 weeks (*P* = 0.012) (Fig. 2b).

Compared to foals without other pleural lesions, the prevalence of respiratory signs started to be significantly higher in foals with other pleural lesions at the age of 7–8 weeks and remained significantly elevated until the foals turned 13–14 weeks old (Fig. 2c).

From the multivariable analysis, we identified two LUS abnormalities significantly and independently linked with the occurrence of respiratory signs—the presence of lung abscess of maximum diameter ≥ 15 mm and the presence of other pleural lesions (Table 2). The presence of at least 1 lung abscess (irrespective of its maximum diameter) and the presence of multiple B-lines turned out to be insignificant in the multivariable analysis. Lung abscesses with a maximum diameter ≥ 15 mm were found in 42/185 foals (22.7%; CI 95% 17.3–29.3%). The age-related prevalence of respiratory signs was significantly higher in foals with lung abscesses of maximum diameter ≥ 15 mm at the age of 3–4 weeks. The age-related prevalence of respiratory signs decreased to a level similar to foals without lung abscesses or with lung abscesses of diameter < 15 mm at the age of 15–16 months (Fig. 2d).

**Table 2:** The mixed-effect binary logistic model evaluating the relationship between the presence of lung ultrasound (LUS) abnormalities and respiratory clinical signs suggestive of *Rhodococcus equi* pneumonia.

Variable	Regression coefficient (standard error)	Model statistic	*P*-value	Adjusted odds ratio (95% confidence intervals)
Fixed effects				
Intercept	− 3.76 (0.64)	–	–	–
Age at examination	–	–	0.497	–
Breed				
Pure Arabian or Polish Anglo-Arabian	Reference category			
Thoroughbred	0.04 (0.79)	0.06	0.957	1.04 (0.22–4.89)
Maximum diameter of the largest lung abscess				
< 15 mm	Reference category	–	–	–
≥ 15 mm	1.96 (0.30)	6.51	< 0.001	7.12 (3.94–12.9)
Other pleural lesions				
None	Reference category	–	–	–
Present	1.69 (0.40)	4.29	< 0.001	5.44 (2.51–11.8)
Variables removed in the backward stepwise elimination				
Number of lung abscesses				
None	Reference category	–	–	–
≥ 1	0.52 (0.51)	1.01	0.312	1.68 (0.61–4.61)
B-lines				
None or single	Reference category	–	–	–
Multiple B lines	0.56 (0.35)	1.60	0.109	1.76 (0.88–3.51)
Random effects				
Foal (F)	0.68 (0.35)	1.96	0.050	–
Stud (S)	0.44 (0.50)	0.89	0.374	–
Year (D)	0.01 (0.13)	0.05	0.962	–

### Diagnostic potential of clinical signs in pulmonary rhodococcosis

Two clinical signs were significantly positively linked to the presence of lung abscesses: body temperature (*P* < 0.001) and the presence and severity of murmurs and crackles in lung auscultation (*P* < 0.001) (Table 3). The AUROC of body temperature was 60.9% (CI 95% 56.5–65.2%), and the optimal cutoff value was ≥ 38.6 °C. At this cutoff value, body temperature had a diagnostic sensitivity (Se) of 38.9% (CI 95% 33.2–45.0) and a diagnostic specificity (Sp) of 79.6% (CI 95% 76.0–82.8%) in predicting the presence of lung abscesses. The presence of mild or marked murmurs and crackles also had low Se (30.9%; CI 95% 25.6–36.8%) but high Sp (89.2%; CI 95% 86.2–91.5%). The positive likelihood ratio (LR+) of mild murmurs was 1.78 (CI 95% 0.70–4.55), while the LR+ of marked murmurs was 7.43 (CI 95% 4.83–11.4). Generally, clinical signs lack sufficient sensitivity to allow for an accurate diagnosis of pulmonary rhodococcosis.

**Table 3:** The mixed linear model (MLM) evaluating the relationship between various clinical signs and the presence of lung abscesses suggestive of *Rhodococcus equi* pneumonia.

Variable	Regression coefficient (standard error)	Model statistic	*P*-value	Adjusted odds ratio (95% confidence intervals)
Fixed effects				
Intercept	− 30.6 (9.11)	–	–	–
Age at examination	− 0.10 (0.05)	− 2.05	0.041	0.90 (0.82–0.996)
Breed				
Arabian (Pure or mixed)	Reference category			
Thoroughbreds	− 0.14 (0.48)	− 0.30	0.765	0.87 (0.34–2.20)
Body temperature (°C)	0.79 (0.24)	3.33	< 0.001	2.20 (1.38–3.50)
Lung auscultation				
Normal	Reference category			
Mild murmurs and crackles	0.89 (0.27)	3.24	0.001	2.43 (1.42–4.16)
Marked murmurs and crackles	1.85 (0.40)	4.63	< 0.001	6.35 (2.90–13.9)
Variables removed in the backward stepwise elimination				
Heart rate per minute	0.01 (0.01)	0.87	0.376	1.01 (0.99–1.02)
Lymph nodes				
Normal	Reference category			
Unilaterally enlarged	0.53 (0.38)	1.38	0.168	1.69 (0.80–3.57)
Bilaterally enlarged	− 0.01 (0.32)	− 0.02	0.987	1.00 (0.53–1.88)
Dyspnea	0.84 (1.25)	0.67	0.501	2.31 (0.20–26.8)
Mucous membranes				
Pink and moist	Reference category			
Pale	− 0.84 (0.93)	− 0.90	0.369	0.43 (0.07–2.69)
Congested	0.95 (1.71)	0.55	0.580	2.58 (0.09–73.9)
Random effects				
Foal (F)	0.83 (0.23)	3.60	< 0.001	–
Stable (S)	0.14 (0.27)	0.54	0.591	–
Year (Y)	0.19 (0.25)	0.75	0.456	–

### Mortality risk

Of 185 foals enrolled in our study, 9 died (5 females, 4 males), yielding a crude mortality of 4.9% (CI 95% 2.6–9.0%). Three foals died unexpectedly with no apparent signs of pneumonia 24 h before death. Additionally, LUS findings during the last examination in these three foals did not indicate an increased risk of death (mild or multiple B-lines detected). Lung consolidations but no abscesses > 10 mm were discovered during these three necropsies. Nevertheless, the bacterial cultures collected from the lungs of all 9 foals lost during necropsies were *R. equi* positive. The remaining 6 foals that were lost had dyspnoea, and rectal body temperature was persistently elevated above 39.5 °C. In 5 of them, lung abscesses increased in size over time, despite the implemented treatment. They had at least one abscess visible on LUS with a maximum diameter > 15 mm and other pleural lesions prior to death. The number of abscesses varied during the monitoring period; however, fusion of the nearest abscesses and multifocal, diffuse pulmonary lesion development seemed common in the most severe cases.

The 3 foals that died unexpectedly had no apparent reasons for sudden death discovered during necropsies. All 3 foals had the last screening examination performed over one week prior to death. Our hypothesis of the most likely cause of death in these cases was acute septicemia due to *R. equi* or a mixed infection (bacterial or viral).

The foals that died belonged to 3 studs; however, 7 foals belonged to the same stud (B). Nonsurvivors were born by 7 mares; two mares in stud B delivered 2 foals that died: one mare in 2019 and 2020 and another mare in 2020 and 2021. The relationship between the month of birth and nonsurvival was significant (p = 0.006): 5/9 (56%) foals that died were born in April (Table S4). However, due to the low number of nonsurvivors and clustering of deaths in only 1 of 5 studs no further conclusions should be made.

## Discussion

Although *R. equi* infection in foals has been considered a serious and widespread problem on endemic farms for decades, data on the field occurrence of rhodococcosis are often incomplete or apply to only one breeding season or a single breeding farm. Our study shows that respiratory clinical signs were observed in over 25% of the foals, whereas approximately 70% of the foals between the 2nd week and 4th month of age developed LUS abnormalities. Respiratory clinical signs were observed significantly (from 2 to 3 to even 6–8 times) more often in foals with LUS abnormalities, including lung abscesses, multiple B-lines, and other pleural lesions. These findings are comparable to those described in Texas^5^ despite different environment and preventive strategies routinely used on breeding farms. Moreover, the age of the first appearance of LUS abnormalities and clinical signs in our study correspond to previously published data, in which a significant decline in rhodococcal colostral antibody levels was observed after the third week of life^22^. To some extent, this finding is in line with earlier studies showing a correlation between the depletion of maternal antibodies and the occurrence of respiratory signs suggestive of rhodococcal pneumonia^23,24^. The peak of LUS abnormalities was observed in 7–8 weeks of life. In our study, a continuous decrease in LUS abnormalities partially correlated with the time of treatment implementation. The severity of respiratory clinical signs remained similar throughout the study, which further supports the low sensitivity of clinical parameters to diagnose *R. equi* pneumonia. In studies by other authors, foals developed clinical signs suggestive of rhodococcal pneumonia and were treated later in life, possibly due to different management practices^8,11,25^.

Our findings only partially agreed with one of the recent and most important results from a breeding farm in Germany that linked only very high TMDs of lung abscesses (up to 15 cm) with treatment implementation^6^. Foals described in another study from Germany^26^ showed clinical signs significantly later than ours (approximately 14–16 weeks of age vs. 7–8 weeks of age, respectively). The age of affected foals appeared to be negatively correlated with the severity of the disease^27^; however, these findings might also be associated with specific management strategies, climate or location and do not necessarily represent general conclusions between the age and severity of the disease. To our knowledge, the morbidity and mortality rates as well as the severity of disease may vary significantly between regions, breeding seasons, or even particular studs^8^. Some of the factors that can explain differences between the situations in studs include the number of foals born per season, the size and construction of the stables, the number of qualified workers, and the availability of pastures^28^. Finally, we consider it difficult to agree with treating only foals presenting both clinical signs and very severe pulmonary lesions, as indicated in one paper^6^, since fever and dyspnoea may be of serious concern, especially in very young foals. Although highly sensitive, LUS also has some limitations in diagnosing pulmonary pathology, and it can visualize abnormalities located close to the pleural surface but not in the pulmonary parenchyma; thus, certain lesions might sometimes be omitted^10,14,29^. Additional diagnostic modalities, such as thoracic radiographs and computed tomography, can also be considered in more complex cases.

According to the results from our study, the presence of LUS abnormalities in foals among Polish studs included in our program did not differ considerably; however, on one of the farms, substantially more foals developed clinical signs. The exact reason for this finding is unknown; however, some of the explanations include different environmental conditions and management strategies. We plan to thoroughly examine this stud in the future. Previous data have shown that despite being a ubiquitous pathogen, virulent *R. equi* may not always be isolated from soil, even on farms with confirmed endemic infections^30–32^. Some authors have indicated the relationship between the density of mares and foals and the increased risk of developing clinical signs^30^. Although there are no strict environmental management practices established for prophylaxis against rhodococcosis, some papers indicate a link between early exposure to airborne *R. equi* and a subsequent higher risk of developing pneumonia^33,34^. The significantly higher prevalence of respiratory signs and mortality rate in stud B further support the effect of the environment on the development of disease. We consider stud B to have worse biosecurity management strategies due to the higher density of horses in one stall and more intense rotation of external mares coming for obstetrical diagnostic and insemination to the stud applied veterinarians. Moreover, studies have shown that both affected foals^35^ and their dams^36^ may have increased faecal shedding of virulent *R. equi*. Insufficient hygienic practices may result in higher levels of so-called alveolar permeable fractions of dust, which are known to reach the lower respiratory tract^37^ and convey some biological particles, such as bacteria^38^. This finding is also compatible with the observation that the air concentration of virulent *R. equi* strains was significantly higher inside the stables than on paddocks^28^. These observations may indicate the influence of environmental factors on the prevalence or course of the disease and partially explain the exacerbation of severe and even fatal cases observed during our study among foals born at the end of the most intense foaling season in Poland. The aforementioned hypothesis is in line with a recent study from Japan, showing an association between the month of birth and initial colonization of *R. equi* in the trachea of neonatal foals, suggesting that a certain period during the foaling season might be positively correlated with developing *R. equi* pneumonia. Moreover, this paper also demonstrates that the greatest percentage of *R. equi-*positive foals based on clinical signs and microbiological culture from transtracheal aspiration detected in foals were born in March and April^39^.

Our study confirmed previous findings that LUS abnormalities suggestive of pulmonary rhodococcosis start to develop from the first weeks of foals’ life, peak around the age of 2 months, and then gradually decline, either with or without treatment. The most common LUS abnormalities are lung abscesses, followed by multiple B-lines and other pleural lesions. Contrary to LUS findings, respiratory clinical signs remained unchanged for the entire period of the first 4 months of foal life. The occurrence of multiple B-lines and other pleural lesions decreased with the resolution of clinical signs, while lung abscesses persisted beyond the first 4 months of life in approximately one-fourth of all foals examined in the study. Our results are in line with previous studies reporting similar routine monitoring of foals for *R. equi* infection^5,7,8,27^.

To some extent, our work supports recently made recommendations to examine the effects of selective antimicrobial treatments^1^. Our results strongly indicated that the presence of pulmonary abscesses with diameters above 15 mm and other pleural lesions are significantly linked with the occurrence of clinical signs of pulmonary rhodococcal infections. They cannot be viewed as the only criteria for treatment implementation but may justify the monitoring of subclinical foals. Unfortunately, the use of LUS has already gained some negative opinions due to excessive antimicrobial treatment of subclinical cases with mild sonographic pulmonary lesions and subsequent alarming growth of antimicrobial resistance^2,3,40–43^. Accurate criteria for recognizing foals with suspected *R. equi* infection that truly need antibiotic therapy are yet to be determined.

Recently, LUS has been shown to be a valid method of recognizing and monitoring pneumonia in preweaned calves^44^. This approach in cattle can be considered similar to the one to rhodococcal pneumonia in foals due to challenges with antemortem diagnosis, field settings, a great number of patients, diversification between farms, and potential disadvantages of other diagnostic tools. LUS examination allows us to monitor the affected animals safely and continuously and to base the decision regarding the introduction and discontinuation of antimicrobial therapy on more precise grounds^45^. This is important in the management of infectious diseases due to possible rapid changes in clinical signs in response to appropriate antibiotic therapy. Early discontinuation of antibiotics in the face of persistent severe pulmonary lesions may lead to the reoccurrence of clinical diseases and significantly worsen the prognosis for survival, especially if antimicrobial resistance has developed^46^. On the other hand, unnecessarily prolonged treatment with antimicrobials may also result in increased antimicrobial resistance; thus, a method of assessing the current status of pneumonia is highly desired^45,46^. This problem has not been frequently investigated in clinical studies, despite the very long duration of recommended therapy. Proper monitoring of clinical signs might allow safe shortening of the treatment, compared to recommended standard protocols, based on LUS findings supporting the decision of discontinuation of the antimicrobials^6,15,26^.

Ultrasound screening has become popular in clinical settings because it can be performed quickly and safely, and the result is immediately available. This is particularly valuable in field conditions, where numerous animals have to be examined in a short time. Laboratory diagnostic methods, such as inflammatory markers (white blood cell counts, fibrinogen concentration, serum amyloid A, or procalcitonin), were not considered accurate factors to recognize the onset and progression of rhodococcosis^47–50^. Additionally, microbiological or PCR confirmation based on methods such as bronchoalveolar lavage (BAL) or tracheobronchial aspirate (TBA) presents serious disadvantages, including the risk of endoscopic examination, which may alter breathing in already dyspnoeic animals or require sedation of the severely ill and pyretic foal^4,51^. Moreover, this method has the potential for false results^52^ and requires some time, which is highly unwanted in regard to neonatal diseases. These concerns especially apply to an early diagnosis in field conditions. Our results, along with some previous studies, support the hypothesis that developing an appropriate ultrasound monitoring program may lead to more effective monitoring of clinical signs and management of pulmonary rhodococcosis in the field condition of a stud. Based on the findings from our study, we propose that performing LUS at least every 2 weeks may improve the diagnosis of the diseases, especially in the most severe cases.

Our study has several limitations, mostly due to its field character and the multiple participants involved. Only two studs participated in the screening for the entire period. Moreover, for organizational reasons, foals were not followed up for the same period, and the number of examinations they underwent was not the same for all of them. This could have influenced the power and reliability of the statistical analysis. Additionally, some procedures needed to be simplified or unified among the participating studs, especially diagnostic procedures. Based on our clinical experience, we decided to shorten the lists of clinical signs and LUS findings connected with pulmonary rhodococcosis to the three most common observations each. Nevertheless, we are aware that the clinical manifestation of rhodococcosis can sometimes differ significantly and depend on the form (pulmonary or extrapulmonary), age of the foal, or severity of LUS findings. Some of our encounters with less typical courses of rhodococcal infections have already been described^53^.

One of the crucial concerns in regard to infectious diseases is the most precise diagnosis. The majority of researchers would prefer to have microbiological or PCR confirmation of the pathogen involved. We agree that such evidence ends most of the discussions about the accuracy of the diagnosis. However, we believe that the risk of misdiagnosis in the case of rhodococcal pneumonia is very limited. The combined clinical and ultrasonographic manifestation of pulmonary rhodococcosis is highly specific. Although not pathognomonic, lung abscessation in the preweaned age is rarely described in cases other than *R. equi*^4,7,51,54^. On the other hand, one study showed that the concentration of virulent *R. equi* in the exhaled air of both healthy and affected foals does not differ significantly^55^. The same results may apply to the material collected during BAL or TBA. The material collected from the trachea and bronchi might be *R. equi* positive in most foals, independent of their health status due to its environmental characteristics. It may result in many false-positive diagnoses. Finally, the most important concern is to limit unnecessary antimicrobial use, but positive results alone of microbiological culture or PCR do not define which foals truly require treatment.

We agree that the results from two (or more) independent veterinarians would add more accuracy to our research; however, no other veterinarian was available to perform such a comparison. Nevertheless, having the same person perform all the diagnostics enabled a high level of consistency. Moreover, the veterinarian who performed LUS was the one most experienced in this procedure on the team, and they were always able to consult with another equine-specialized veterinarian regarding the results.

Owing to the field character of this study, it may be considered highly representative of the actual health status and decisions regarding rhodococcosis. However, its character may also impose a limit to some of the analysis and results. We expect the implementation of antimicrobial treatment to have a substantial impact on the presence of clinical findings and lung abnormalities^15^. In most cases, implementing antimicrobial drugs allowed us to reduce fever and improve clinical conditions within a few days. Moreover, the size of most abscesses also gradually decreased after treatment implementation. Unfortunately, we cannot be sure about the extent of the effect of antimicrobial therapy since we did not compare treated foals with any foals given a placebo. Such a comparison would certainly be highly valuable from an academic point of view, but we consider such a study unethical. A paper considering some of the effects of different drugs or their combinations, including the placebo group, was already published; however, the study was performed on foals with lung abscesses and elevated WBC, regardless of their clinical status^3^. Moreover, changes in the therapeutic plan were regulated in accordance with the current health status of a particular foal and often modified. The relationship between the size of the abscesses and the presence of clinical findings in our study might be partially influenced by these variabilities; however, these limitations would be very hard to eliminate.

Additionally, some unfortunate circumstances, including widespread abortions due to the equine herpes virus outbreak in 2019 and limitations in travelling in 2020/2021 due to the COVID-19 pandemic, substantially reduced the number of participating foals. Finally, as university workers, we were responsible for repeated clinical and ultrasound examinations according to the LUS monitoring program. While we could recommend additional diagnostic procedures and treatment options, the decisive voice belonged to the owners and stud veterinarians. In the case of field clinical research on quite expensive animals, the consequences of invasive diagnostic procedures and different treatment approaches were also vastly considered.

## Conclusions

In Poland, clinical pulmonary rhodococcosis affects approximately 1/4 of the population of foals in some selected endemic studs, while subclinical pulmonary rhodococcosis appears to be present in approximately 70% of foals in these studs. The most frequently observed LUS abnormalities in preweaned foals are lung abscesses occurring at approximately 2 months of age. Other pleural lesions and multiple B-lines were less frequent. The presence of lung abscesses of a maximum diameter larger than 15 mm and the presence of other pleural lesions are significantly and strongly linked with the occurrence of clinical rhodococcosis, so they may be considered factors indicating the development of clinical disease. Increased body temperature and the presence of wheezes and crackles in chest auscultation are significantly associated with the presence of lung abscesses; however, clinical signs lack sufficient diagnostic sensitivity to allow for an accurate diagnosis of rhodococcosis. Future studies are needed to develop more efficient monitoring and treatment protocols in equine breeding farms.

### Supplementary Information

Supplementary Figure S1.

Supplementary Tables.

Supplementary Table S3.

Supplementary Table S4.

## Data Availability

Any additional information might be available on request from the corresponding author.

## References

[1] Giguère S, Cohen ND (2018). Controversies in therapy of infections caused by *Rhodococcus equi* in foals. Equine Vet. Educ..

[2] McCracken, J. L. Screening for *Rhodococcus equi* pneumonia. in *Robinson’s Current Therapy in Equine Medicine*, 737–740 (Elsevier, 2015). 10.1016/B978-1-4557-4555-5.00176-X.

[3] Venner M, Rödiger A, Laemmer M, Giguère S (2012). Failure of antimicrobial therapy to accelerate spontaneous healing of subclinical pulmonary abscesses on a farm with endemic infections caused by *Rhodococcus equi*. Vet. J..

[4] Huber L (2018). Monitoring foals by thoracic ultrasonography, bacterial culture, and PCR: Diagnostic of *Rhodococcus equi* subclinical pneumonia in south of Brazil. J. Equine Vet. Sci..

[5] Madrigal RG (2016). Use of serial quantitative PCR of the vapA gene of Rhodococcus equi in feces for early detection of *R. equi* pneumonia in foals. J. Vet. Intern. Med..

[6] Arnold-Lehna D, Venner M, Berghaus LJ, Berghaus R, Giguère S (2019). Changing policy to treat foals with *Rhodococcus equi* pneumonia in the later course of disease decreases antimicrobial usage without increasing mortality rate. Equine Vet. J..

[7] McCracken JL, Slovis NM (2009). Use of thoracic ultrasound for the prevention of *Rhodococcus equi* pneumonia on endemic farms. AAEP Proc..

[8] Coleman MC (2019). Foal-level risk factors associated with development of *Rhodococcus equi* pneumonia at a quarter horse breeding farm. J. Equine Vet. Sci..

[9] Tarancón I, Leiva M, Jose-Cunilleras E, Ríos J, Peña T (2019). Ophthalmologic findings associated with *Rhodococcus equi* bronchopneumonia in foals. Vet. Ophthalmol..

[10] Venner M, Walther SM, Münzer B, Stadler P (2014). Diagnostic of Pulmonary Abscesses in foals-Comparison of Sonographic and Radiographic Examination.

[11] Kahn SK (2021). Randomized, controlled trial comparing *Rhodococcus equi *and poly-N-acetyl glucosamine hyperimmune plasma to prevent *R. equi* pneumonia in foals. J. Vet. Intern. Med..

[12] Kalinowski M, Jarosz Ł, Grądzki Z (2020). Assessment of antimicrobial susceptibility of virulent strains of *Rhodococcus equi* isolated from foals and soil of horse breeding farms with and without endemic infections. J. Equine Vet. Sci..

[13] Giguère S (2011). Diagnosis, treatment, control, and prevention of infections caused by *Rhodococcus equi* in foals. J. Vet. Intern. Med..

[14] Łobaczewski A (2021). Lung ultrasound for imaging of b-lines in dogs and cats: A prospective study investigating agreement between three types of transducers and the accuracy in diagnosing cardiogenic pulmonary edema, pneumonia and lung neoplasia. Animals.

[15] Rutenberg D, Venner M, Giguère S (2017). Efficacy of tulathromycin for the treatment of foals with mild to moderate bronchopneumonia. J. Vet. Intern. Med..

[16] Giguére S (2004). Retrospective comparison of azithromycin, clarithromycin, and erythromycin for the treatment of foals with *Rhodococcus equi* pneumonia. J. Vet. Intern. Med..

[17] Rzewuska M (2014). Characterization of *Rhodococcus equi* isolates from submaxillary lymph nodes of wild boars (*Sus scrofa*), red deer (*Cervus elaphus*) and roe deer (*Capreolus capreolus*). Vet. Microbiol..

[18] Traczewski MMBM (2017). Methods for Antimicrobial Susceptibility Testing of Infrequently Isolated or Fastidious Bacteria Isolated From Animals.

[19] Altman D, Machin D, Bryant T, Gardner M (2000). Statistics with Confidence: Confidence Intervals and Statistical Guidelines.

[20] Chaffin MK, Cohen ND, Martens RJ (2003). Evaluation of equine breeding farm management and preventative health practices as risk factors for development of Rhodococcus equi pneumonia in foals. J. Am. Vet. Med. Assoc..

[21] Cohen ND, O’Conor MS, Chaffin MK, Martens RJ (2005). Farm characteristics and management practices associated with development of *Rhodococcus equi* pneumonia in foals. J. Am. Vet. Med. Assoc..

[22] Studies on the persistence of transfused *Rhodococcus equi* antibodies in Thoroughbred foals. *Anim. Reprod. Sci.* **121**, 359–360 (2010).

[23] Lopez AM, Hines MT, Palmer GH, Alperin DC, Hines SA (2002). Identification of pulmonary T-lymphocyte and serum antibody isotype responses associated with protection against *Rhodococcus equi*. Clin. Diagn. Lab. Immunol..

[24] Sanz MG, Loynachan A, Horohov DW (2016). *Rhodococcus equi* hyperimmune plasma decreases pneumonia severity after a randomised experimental challenge of neonatal foals. Vet. Rec..

[25] Kahn SK (2019). Transfusion with 2 L of hyperimmune plasma is superior to transfusion of 1 L or less for protecting foals against subclinical pneumonia attributed to *Rhodococcus equi*. J. Equine Vet. Sci..

[26] Wetzig M, Venner M, Giguère S (2019). Efficacy of the combination of doxycycline and azithromycin for the treatment of foals with mild to moderate bronchopneumonia. Equine Vet. J..

[27] Arnold-Lehna D, Venner M, Berghous LJ, Berghous R, Giguére S (2019). Efficacy of treatment and survival rate of foals with pneumonia: Retrospective comparison of rifampin/azithromycin and rifampin/tulathromycin. Pferdeheilkunde.

[28] Muscatello G (2010). Comparison of concentrations of *Rhodococcus equi* and virulent *R. equi* in air of stables and paddocks on horse breeding farms in a temperate climate. Equine Vet. J..

[29] Punsmann S, Hellige M, Hoppe J, Freise F, Venner M (2021). Diagnostic imaging in acute interstitial pneumonia in foals: High variability of interpretation of chest radiographs and good conformity between ultrasonographic and post-mortem findings. Vet. Radiol. Ultrasound.

[30] Cohen ND (2008). Association of soil concentrations of *Rhodococcus equi* and incidence of pneumonia attributable to *Rhodococcus equi* in foals on farms in central Kentucky. Am. J. Vet. Res..

[31] Takai S (2001). Prevalence of virulent *Rhodococcus equi* in soil from five *R. equi*-endemic horse-breeding farms and restriction fragment length polymorphisms of virulence plasmids in isolates from soil and infected foals in Texas. J. Vet. Diagn. Invest..

[32] Martens RJ (2000). Association of disease with isolation and virulence of *Rhodococcus equi* from farm soil and foals with pneumonia. J. Am. Vet. Med. Assoc..

[33] Cohen ND (2013). Association of perinatal exposure to airborne *Rhodococcus equi* with risk of pneumonia caused by *R. equi* in foals. Am. J. Vet. Res..

[34] Kuskie KR (2011). Associations between the exposure to airborne virulent *Rhodococcus equi* and the incidence of *R. equi* pneumonia among individual foals. J. Equine Vet. Sci..

[35] Witkowski L (2017). Molecular characterization of *Rhodococcus equi* isolates from horses in Poland: PVapA characteristics and plasmid new variant, 85-kb type V. BMC Vet. Res..

[36] Huber L, Giguère S, Berghaus LJ, Hanafi A, Ryan C (2018). Fecal shedding of *Rhodococcus equi* in mares and foals after experimental infection of foals and effect of composting on concentrations of *R. equi* in contaminated bedding. Vet. Microbiol..

[37] Claußen G, Hessel EF (2017). Particulate matter in equestrian stables and riding arenas. J. Equine Vet. Sci..

[38] Grzyb J, Podstawski Z, Bulski K (2021). Bacterial aerosol, particulate matter, and microclimatic parameters in the horse stables in Poland. Environ. Sci. Pollut. Res..

[39] Takai S (2022). Birth month associated with tracheal colonization of *Rhodococcus equi* in newborn foals on horse-breeding farms with sporadic rhodococcosis in Japan. Vet. Microbiol..

[40] Giguère S, Berghaus LJ, Willingham-Lane JM (2017). Antimicrobial resistance in *Rhodococcus equi*. Vet. Microbiol..

[41] Erol E, Shaffer CL, Lubbers BV (2021). Synergistic combinations of clarithromycin with doxycycline or minocycline reduce the emergence of antimicrobial resistance in *Rhodococcus equi*. Equine Vet. J..

[42] Huber L (2018). Emergence of resistance to macrolides and rifampin in clinical isolates of *Rhodococcus equi* from foals in Central Kentucky, 1995 to 2017. Antimicrob. Agents Chemother..

[43] Huber L (2019). Prevalence and risk factors associated with emergence of *Rhodococcus equi* resistance to macrolides and rifampicin in horse-breeding farms in Kentucky, USA. Vet. Microbiol..

[44] Rhodes V (2021). Diagnosis of respiratory disease in preweaned dairy calves using sequential thoracic ultrasonography and clinical respiratory scoring: Temporal transitions and association with growth rates. J. Dairy Sci..

[45] Jourquin S (2022). Randomized field trial comparing the efficacy of florfenicol and oxytetracycline in a natural outbreak of calf pneumonia using lung reaeration as a cure criterion. J. Vet. Intern. Med..

[46] Palma E, Tilocca B, Roncada P (2020). Antimicrobial resistance in veterinary medicine: An overview. Int. J. Mol. Sci..

[47] Giguère S, Berghaus LJ, Miller CD (2016). Clinical assessment of a point-of-care serum amyloid A assay in foals with bronchopneumonia. J. Vet. Intern. Med..

[48] Cohen ND (2010). Study of serum amyloid A concentrations as a means of achieving early diagnosis of *Rhodococcus equi* pneumonia. Equine Vet. J..

[49] Passamonti F (2015). *Rhodococcus equi* pneumonia in foals: An assessment of the early diagnostic value of serum amyloid A and plasma fibrinogen concentrations in equine clinical practice. Vet. J..

[50] Giguère S, Hernandez J, Gaskin J, Miller C, Bowman JL (2003). Evaluation of white blood cell concentration, plasma fibrinogen concentration, and an agar gel immunodiffusion test for early identification of foals with *Rhodococcus equi* pneumonia. J. Am. Vet. Med. Assoc..

[51] Leclere M, Magdesian KG, Kass PH, Pusterla N, Rhodes DM (2011). Comparison of the clinical, microbiological, radiological and haematological features of foals with pneumonia caused by *Rhodococcus equi* and other bacteria. Vet. J..

[52] Hillidge CJ (1987). Use of erythromycin-rifampin combination in treatment of *Rhodococcus equi* pneumonia. Vet. Microbiol..

[53] Rakowska A (2022). Less typical courses of *Rhodococcus equi*infections in foals. Vet. Sci..

[54] Bianchi MV (2020). Causes and pathology of equine pneumonia and pleuritis in southern Brazil. J. Comp. Pathol..

[55] Muscatello G, Gilkerson JR, Browning GF (2009). Detection of virulent *Rhodococcus equi* in exhaled air samples from naturally infected foals. J. Clin. Microbiol..

