# Supplementary material for: Investigation of the relationship between pulmonary lesions based on lung ultrasound and respiratory clinical signs in foals with suspected pulmonary rhodococcosis
Source: Sci Rep. 2023 Nov 8;13:19401. doi: 10.1038/s41598-023-46833-2 (PMC10632467; doi:10.1038/s41598-023-46833-2)
Supplement: Supplementary file 1 — Supplementary Figure S1. [file 41598_2023_46833_MOESM1_ESM.docx]

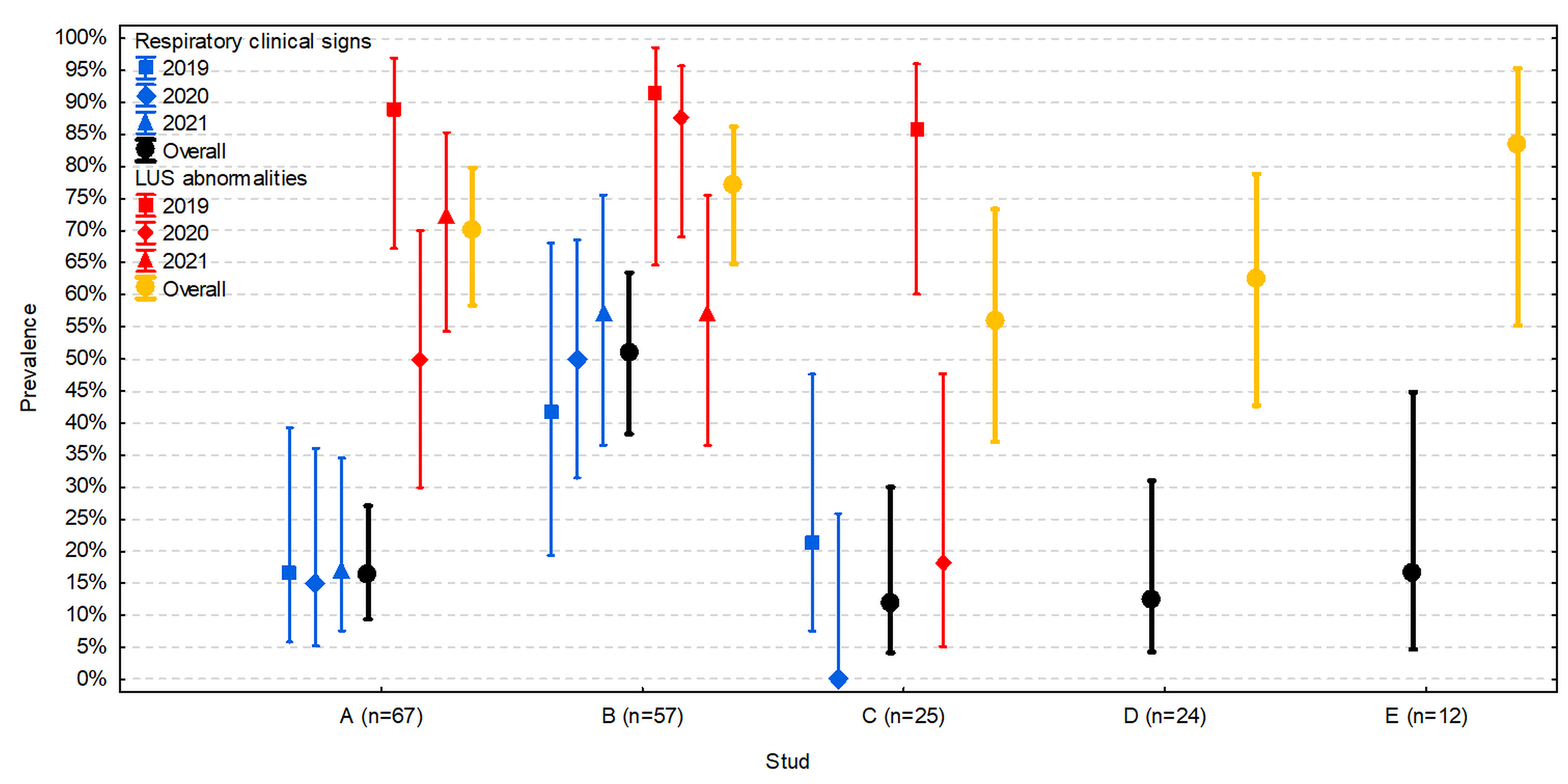


Fig. S1. Prevalence of foals with respiratory clinical signs and lung ultrasound (LUS) abscesses in 5 studs in which the study was carried out split into 3 years of the study
