# Supplementary material for: Investigation of the relationship between pulmonary lesions based on lung ultrasound and respiratory clinical signs in foals with suspected pulmonary rhodococcosis
Source: Sci Rep. 2023 Nov 8;13:19401. doi: 10.1038/s41598-023-46833-2 (PMC10632467; doi:10.1038/s41598-023-46833-2)
Supplement: Supplementary file 2 — Supplementary Tables. [file 41598_2023_46833_MOESM2_ESM.docx]

Table S1. The number of foals examined a particular number of times

| No. of times examined | No. of foals |
| --- | --- |
| 1 | 26 (14.1) |
| 2 | 17 (9.2) |
| 3 | 18 (9.7) |
| 4 | 17 (9.2) |
| 5 | 43 (23.2) |
| 6 | 48 (25.9) |
| 7 | 26 (14.1) |

Table S2. The number of foals examined at each age

| Age at which examined [weeks] | No. of foals | % of 185 | Females | Males |
| --- | --- | --- | --- | --- |
| 1-2 | 4 | 2.1 | 2 | 2 |
| 3-4 | 93 | 49.2 | 55 | 38 |
| 5-6 | 123 | 65.1 | 70 | 53 |
| 7-8 | 131 | 69.3 | 69 | 62 |
| 9-10 | 135 | 71.4 | 71 | 64 |
| 11-12 | 135 | 71.4 | 72 | 63 |
| 13-14 | 122 | 64.6 | 61 | 61 |
| 15-16 | 54 | 28.6 | 28 | 26 |
