# Supplementary material for: Investigation of the relationship between pulmonary lesions based on lung ultrasound and respiratory clinical signs in foals with suspected pulmonary rhodococcosis
Source: Sci Rep. 2023 Nov 8;13:19401. doi: 10.1038/s41598-023-46833-2 (PMC10632467; doi:10.1038/s41598-023-46833-2)
Supplement: Supplementary file 3 — Supplementary Table S3. [file 41598_2023_46833_MOESM3_ESM.docx]

Table S3. Age-related prevalence of respiratory clinical signs and lung ultrasound (LUS) abnormalities in foals enrolled in the study

| Age at which examined [weeks] | No. of foals examined | Fever (>39.5°C) | Marked murmur or crackles on auscultation | Dyspnea | Any respiratory clinical signs | | Lung abscesses | | Multiple B-lines | | Pleural lesions | | Any LUS abnormalities | |
| --- | --- | --- | --- | --- | --- | --- | --- | --- | --- | --- | --- | --- | --- | --- |
|  |  |  |  |  | n | Prevalence (CI 95%) | n | Prevalence (CI 95%) | n | Prevalence (CI 95%) | n | Prevalence (CI 95%) | n | Prevalence (CI 95%) |
| 1-2 | 4 | 0 | 0 | 0 | 0 | 0 | 0 | 0 | 0 | 0 | 0 | 0 | 0 | 0 |
| 3-4 | 93 | 4 (4.3) | 5 (5.4) | 0 | 6 | 6.5 (3.0 – 13.4) | 17 | 18.3 (11.7 – 27.3) | 5 | 5.4 (2.3 – 12.0) | 1 | 1.1 (0.2 – 5.8) | 18 | 19.4 (12.6 – 28.5) |
| 5-6 | 123 | 6 (4.9) | 2 (1.6) | 0 | 8 | 6.5 (3.3 – 12.3) | 50 | 40.7 (32.4 – 49.5) | 18 | 14.6 (9.5 – 21.9) | 4 | 3.3 (1.3 – 8.1) | 59 | 48.0 (39.3 – 56.7) |
| 7-8 | 131 | 11 (8.4) | 12 (9.2) | 3 (2.3) | 17 | 13.0 (8.3 – 19.8) | 60 | 45.8 (37.5 – 54.3) | 36 | 27.5 (20.6 – 35.7) | 17 | 13.0 (8.3 – 19.8) | 78 | 59.5 (51.0 – 67.6) |
| 9-10 | 135 | 8 (5.9) | 13 (9.6) | 2 (1.5) | 15 | 11.1 (6.8 – 17.5) | 54 | 40.0 (32.1 – 48.4) | 23 | 17.0 (11.6 – 24.3) | 13 | 9.6 (5.7 – 15.8) | 66 | 48.9 (40.6 – 57.2) |
| 11-12 | 135 | 6 (4.4) | 10 (7.4) | 1 (0.7) | 14 | 10.4 (6.3 – 16.7) | 34 | 25.2 (18.6 – 33.1) | 19 | 14.1 (9.2 – 20.9) | 11 | 8.1 (4.6 – 14.0) | 48 | 35.6 (28.0 – 43.9) |
| 13-14 | 122 | 4 (3.3) | 7 (5.7) | 2 (1.6) | 11 | 9.0 (5.1 – 15.4) | 33 | 27.0 (20.0 – 35.5) | 15 | 12.3 (7.6 – 19.3) | 8 | 6.6 (3.4 – 12.4) | 43 | 35.2 (27.3 – 44.1) |
| 15-16 | 54 | 2 (3.7) | 2 (3.7) | 1 (1.9) | 4 | 7.4 (2.9 – 17.6) | 14 | 25.9 (16.1 – 38.9) | 7 | 13.0 (6.4 – 24.4) | 1 | 1.9 (0.3 – 9.8) | 18 | 33.3 (22.2 – 46.6) |
