# Supplementary material for: Investigation of the relationship between pulmonary lesions based on lung ultrasound and respiratory clinical signs in foals with suspected pulmonary rhodococcosis
Source: Sci Rep. 2023 Nov 8;13:19401. doi: 10.1038/s41598-023-46833-2 (PMC10632467; doi:10.1038/s41598-023-46833-2)
Supplement: Supplementary file 4 — Supplementary Table S4. [file 41598_2023_46833_MOESM4_ESM.docx]

Table S4. Distribution of births of survivors and non-survivors in months

| Month of birth | Survived | Died | Studs on which deaths took place |
| --- | --- | --- | --- |
| January | 16 | 2 | B - 2 |
| February | 41 | 0 |  |
| March | 48 | 1 | B – 1 |
| April | 30 | 5 | A – 1; B – 3; C – 1 |
| May | 39 | 0 |  |
| June | 1 | 1 | B – 1 |
| July | 1 | 0 |  |
| Total | 176 | 9 |  |
